# Clinicopathological features and differential diagnosis of gastric pleomorphic giant cell carcinoma

**DOI:** 10.1515/biol-2022-0683

**Published:** 2023-09-11

**Authors:** Yang-Kun Wang, Li Ma, Zhi-Qiang Wang, Yue Wang, Ping Li, Bo Jiang, Su-Nan Wang

**Affiliations:** Department of Pathology, The Fourth People’s Hospital of Longgang District, Shenzhen 518123, China; Clinical Laboratory Department of the 989th Hospital of the PLA Joint Logistics Support Force, Luoyang 471031, China; Department of Pathology, Foresea Life Insurance Guangzhou General Hospital, Guangzhou 511300, China; Shenzhen Hezheng Hospital, Shenzhen 518053, China; Department of Pathology, Peking University Shenzhen Hospital, Shenzhen 518036, China; Department of Pathology, No. 990 Hospital of the PLA Joint Logistics Support Force, Zhumadian 463000, China; Shenzhen Polytechnic, Xili Lake, Xilihu Town, Nanshan District, Shenzhen 518055, China

**Keywords:** gastric tumors, pleomorphic giant cell carcinoma, histomorphology, HER2 gene, immunohistochemistry, differential diagnosis

## Abstract

The aim of this study was to investigate the clinicopathological features and differential diagnosis of gastric pleomorphic giant cell carcinoma. Histopathology, immunohistochemistry, and human epidermal growth factor receptor 2 (HER2) gene testing were conducted for seven cases of gastric pleomorphic giant cell carcinoma. In histomorphological terms, all seven cases involved pleomorphic giant cell carcinoma, accounting for more than 10% of the entire tumor, with pleomorphic spindle cells and giant cells mixed with various histomorphological structures of adenocarcinoma with high, intermediate, and low differentiation. There was large heterogeneity in the HER2 protein expression and HER2 gene amplification in the gastric pleomorphic giant cell carcinoma, and both levels of HER2 were focal in three cases, accounting for 42.9% (3/7). The mismatch repair gene proteins MLH1, MSH2, PMS2, and MSH6 were positive. Routine immunohistochemical markers, i.e., pan-cytokeratin, epithelial membrane antigen, villin, caudal-type homeobox 2, E-cadherin, and p53, were positive in the gastric pleomorphic giant cell carcinoma, while vimentin, calponin, smooth muscle actin, nestin, S-100, cluster of differentiation (CD) 99, desmin, and CD34 were focally expressed in both the spindle and the giant cells, with Ki-67-positive cells accounting for 70–80%. Gastric pleomorphic giant cell carcinoma presents multiple histomorphological features and is easily confused with various tumors. Clarifying the histopathological features of this type of tumor is important for differential diagnosis and precise treatment.

## 1 Contribution to the field

Our study found that gastric pleomorphic giant cell carcinoma is rare. In this study, based on the morphological and histological characteristics of cancer cells, gastric pleomorphic giant cell carcinoma is defined as: pleomorphic spindle cells and giant cell components occupy the entire more than 10% of the tumor, while the pleomorphic spindle cell and giant cell components and any proportion of highly, moderately, and poorly differentiated adenocarcinoma components of a variety of histological and morphological structures are mixed and arranged in a staggered manner. Gastric pleomorphic giant cell carcinoma in addition to the pleomorphism of spindle cells, especially, emphasizes the pleomorphism of giant cells and has small, medium, and large giant cell types and morphological characteristics. Gastric pleomorphic giant cell carcinoma has a variety of histomorphological features and is easily confused with a variety of tumors; clarifying the histopathological features of the tumor is of great significance for differential diagnosis and precise treatment.

## 2 Introduction

Pleomorphic giant cell carcinoma, a variant of pancreatic cancer first proposed by Sommers and Meissner in 1954 [1], exhibits a sarcomatoid transformation characterized by a mixed morphology of undifferentiated cells with significant differences in terms of size and shape [2]. In previous studies, pleomorphic giant cancer cells have been found to have cancer stem cell properties that repopulate tumors and promote breast cancer progression, metastasis, and recurrence through modulating the tumor microenvironment, thus affecting the prognosis and survival of patients with breast cancer [3,4]. In recent years, pleomorphic giant cell carcinoma has been increasingly reported in a large number of retrospective case studies, involving the imaging and clinicopathological features of pleomorphic giant cell carcinoma of the lung [5], thyroid [6], pancreas [7], bladder [8], prostate [9], and rectum [10] as well as the prognostic value of pleomorphic giant cancer cells and the for neoadjuvant radiotherapy. However, gastric pleomorphic giant cell carcinoma was less discussed.

In 2008, Caruso et al. [11] reported the histomorphological features of a 70-year-old male patient with gastric pleomorphic giant cell carcinoma, characterized by aggregates of mononuclear or multinucleated giant cells, extensive coagulative necrosis, and numerous mitoses. It is highly heterogeneous in terms of the occurrence, recurrence, and metastasis, along with the histomorphology, immunophenotype, DNA ploidy, and molecular biological and genetic features [12,13,14,15]. Meanwhile, the efficacy of neoadjuvant chemotherapy and the prognostic indicators of gastric cancer patients with pleomorphic giant cell carcinoma were heterogeneous as well [16]. Our previous study found that the stomach had clinicopathological features of mucinous differentiated adenocarcinoma, exhibiting significantly different prognosis-basing ratios of mucinous components and different histological types of cancer cell components [17]. Furthermore, the clinicopathological features of gastric fibromatous undifferentiated carcinoma [18] also suggest the importance of histopathological staging for precise treatment and prognostic assessment.

In the present study, the data of seven cases of gastric pleomorphic giant cell carcinoma are collected, and the histopathological features, diagnosticfeatures, immunophenotypes, human epidermal growth factor receptor 2 (HER2) gene expression, and differential diagnosis are assessed to enhance the understanding of this type of tumor.

## 3 Materials and methods

### 3.1 Clinical data

Seven cases of gastric pleomorphic giant cell carcinoma of the stomach and esophagogastric junction were collected, which were surgically resected from June 2020 to December 2021 at Foresea Life Insurance Guangzhou General Hospital, the Shenzhen Hospital of Peking University, and the 989 Hospital of PLA Joint Logistic Support Force. The diagnostic criteria were based on the histological classification of gastric cancer presented in *WHO Classification of Digestive Tumors* (2019 edition) [19]. All seven cases were classified as gastric pleomorphic carcinoma, with 4 males and 3 females (age 46–76 years, mean age is 62.4 years), respectively.


**Informed consent:** Informed consent has been obtained from all individuals included in this study.
**Ethical approval:** The research related to human use has been complied with all the relevant national regulations, institutional policies, and in accordance with the tenets of the Helsinki Declaration, and has been approved by Ethics Committee of 989th Hospital of the PLA Joint Logistics Support Force.

### 3.2 Methods

All collected specimens were fixed with 10% neutral buffered formalin within 30 min and freshly prepared for 8–48 h after being surgically removed from the body, with a fixative solution/tissue volume ratio of 10:1. The tissues in the tumor areas were fully and conventionally cut according to the depth of invasion and different colors and textures. Four to six tissue specimens were excised from the central and peripheral areas of the tumor (the tissue samples were routinely excised from the proximal and distal cut edges, while tissue specimens from the tumor and the adjacent gastric mucosa were excluded). One tissue sample from the deepest infiltration point and the most adjacent plasma layer and various tissue samples from all lymph nodes and cancer nodes were excised by subdivision. The hematoxylin–eosin staining (HE), immunohistochemistry (IHC), and genetic testing were performed separately.

### 3.3 IHC

#### 3.3.1 Routine immunohistochemical staining

Here, the EnVision two-step method was used. Pan-cytokeratin (CKpan), carcinoembryonic antigen (CEA), CDX2, villin, SMA, Calponin, vimentin, tumor suppressor protein p53, and Ki-67 were selected as the primary antibodies alongside DNA mismatch repair (MMR) protein assays for MLH1, MSH2, PMS2, and MSH6. The corresponding kits mentioned above were purchased from Shenzhen Dartmon Biotechnology Co., Ltd., and the operations were performed strictly according to the kits’ instructions.

### 3.4 Programmed death-1/programmed death ligand-1/HER2 immunohistochemical assay

#### 3.4.1 Reagents and staining methods

For the programmed death ligand-1 (PD-L1; 22C3), programmed death-1 (PD1; 2E5), and HER2 (SP3) testing, the EnVision method was used, with the procedure performed strictly according to the provided instructions. Here, phosphate-buffered saline was used instead of a primary antibody as the negative control, while placental villi and lymph nodes were used as the positive controls for the PD-L1 and PD1 testing, respectively. The ready-to-use antibody kit and the primary antibody used were purchased from MXB Biotechnologies.

#### 3.4.2 Determination of the programmed death-1/programmed death ligand-1/HER2 results

Concerning the PD-L1 proportional score and the tumor proportion score of the tumor cells, the latter relates to the percentage of tumor cells with partial or complete membrane staining (≥1+) among all live tumor cells (negative and positive) in a sample. The PD-L1 score includes 100 viable infiltrating tumor cells, while the staining intensity of the non-specific background being less than 1+. The tumor stromal lymphocytes are PD-1 positive when located in the cell membrane and/or cytoplasm, appearing as brownish-brown color, which is determined as positive [20].

ISH was performed in cases with IHC 2+ at diagnosis and in all available cases at the time of analysis. HER2 positivity was defined as IHC 3+, or as IHC 2+ with HER2 gene amplification by ISH. Loss of HER2 positivity was defined as IHC score 0/1+, or as IHC 2+ with no amplification by ISH on post-progression biopsies [21,22,23,24].

Meanwhile, referring to determining a positive staining area, if the membranes of the tumor cells were not stained, the area was deemed negative; if over 80% of the area was stained, it was deemed to be a generalized type. If 21–79% of the area was stained, it was deemed a partial type, and if ≤20% of the area was stained, it was deemed a focal type. The results were read by two pathologists blinded to all other information.

### 3.5 Fluorescence *in situ* hybridization (FISH) assay

#### 3.5.1 Reagents, probes, and FISH procedure

The Paraffin Pretreatment Kit II (primarily comprising pretreatment solution and protease solution) and PathVysion™ HER-2 Probe Kit were purchased from Vysis Corporation. The pretreatment procedure and FISH operation steps for the paraffin-embedded gastric cancer tissue sections were performed following previously described procedures [25,26,27] and the kit instructions.

#### 3.5.2 Determination of FISH results

The HER2-positive area of a section was detected via IHC staining using the FISH technique. First, the positive area of gastric adenocarcinoma cells was identified on the HE-stained section, the same visual field of HE was found on the FISH section under a 10× objective lens, and the whole section was observed under a 40× objective lens. If the nuclei of more than 75% of the cancer cells exhibited hybridization signals, the results were satisfactory. At least 30 cancer cells that had intact boundaries and were isolated and non-overlapped were counted under a 100× objective lens.

HER2 gene amplification was evaluated by counting signals in the 20 non-overlapping tumor cells with the highest gene counts. ISH was interpreted as positive if the HER2/CEP17 ratio was >2.2 and negative if the HER2/CEP17 ratio was <1.8.

When the results were equivocal (1.8–2.2), 20 additional tumor cells were counted, and an HER2/CEP17 ratio of ≥2 was considered positive. Based on the criteria of genetic heterogeneity in HER2-amplified breast cancer, genetic heterogeneity was defined as 5 to <50% of tumor nuclei with an HER2/CEP17 ratio of ≥2.0 by ISH. Here, gene amplification was classified into cluster amplification, large granular amplification, and dot amplification.

### 3.6 Statistical analysis

All data were statistically analyzed using the SPSS22 software (Armonk, NY: IBM Corp.) A *P-*value of <0.05 was considered statistically significant.

### 3.7 Follow-up

The follow-up ended on January 31, 2022, for the subjects enrolled from June 2020 to December 2021 over the phone or via postal communication with the patients or their families.

## 4 Results

### 4.1 Clinical and macroscopic features

The population sample included four males and three females with a mean age of 62.4 years. There were four cases of tumors of the antrum and three cases of lesser curvature of gastric body tumors. All tumors are flat-type lesions (0-Ⅱ): including flat-type (0-Ⅱb+Ⅱc) in 3 cases, and mild depressed-type (0-Ⅱc + Ⅱa) in 2 cases. The tumor diameters ranged from 4.5 to 21.3 cm, with a mean diameter of 7.2 cm. The tumors had no capsule, exhibited infiltrative growth, were soft in texture, and were a gray-white color at the cut surface. In terms of pathological staging, there were one case of pT2N0Mx, one case of pT3N1Mx, two cases of pT3N2Mx, and three cases of pT4aN1Mx (Table 1).

**Table 1:** Clinicopathologic features and follow-up results of pleomorphic giant cell carcinoma of stomach

	Age	Gender	Lesion site	Borrmann classification	Operation	Pathologic Diagnosis	pTNM	Follow-up time (month)	Follow-up results
1	54	M	Antrum	Ⅲ	Partial gastrectomy	Pleomorphic giant cell carcinoma of stomach	pT3N2Mx	11	No recurrence
					Roux-en-Y (R-Y) gastrojejunostomy				
2	76	F	Antrum	Ⅲ	Partial gastrectomy	Pleomorphic giant cell carcinoma of stomach	pT4aN1Mx	14	No recurrence
					Roux-en-Y (R-Y) gastrojejunostomy				
3	68	M	Lesser curvature	Ⅲ	Partial gastrectomy	Pleomorphic giant cell carcinoma of stomach	pT4aN1Mx	6	No recurrence
					Roux-en-Y (R-Y) gastrojejunostomy				
4	66	F	Antrum	Ⅲ	Partial gastrectomy	Pleomorphic giant cell carcinoma of stomach	pT4aN1Mx	10	No recurrence
					Roux-en-Y (R-Y) gastrojejunostomy				
5	55	M	Lesser curvature	Ⅲ	Partial gastrectomy	Pleomorphic giant cell carcinoma of stomach	pT3N2Mx	15	No recurrence
					Roux-en-Y (R-Y) gastrojejunostomy				
6	72	M	Lesser curvature	Ⅲ	Partial gastrectomy	Pleomorphic giant cell carcinoma of stomach	pT3N1Mx	9	No recurrence
					Roux-en-Y (R-Y) gastrojejunostomy				
7	46	F	Antrum	Ⅱ	Partial gastrectomy	Pleomorphic giant cell carcinoma of stomach	pT2N0Mx	18	No recurrence
					Roux-en-Y (R-Y) gastrojejunostomy				

### 4.2 Histopathological features

**Figure 1:**
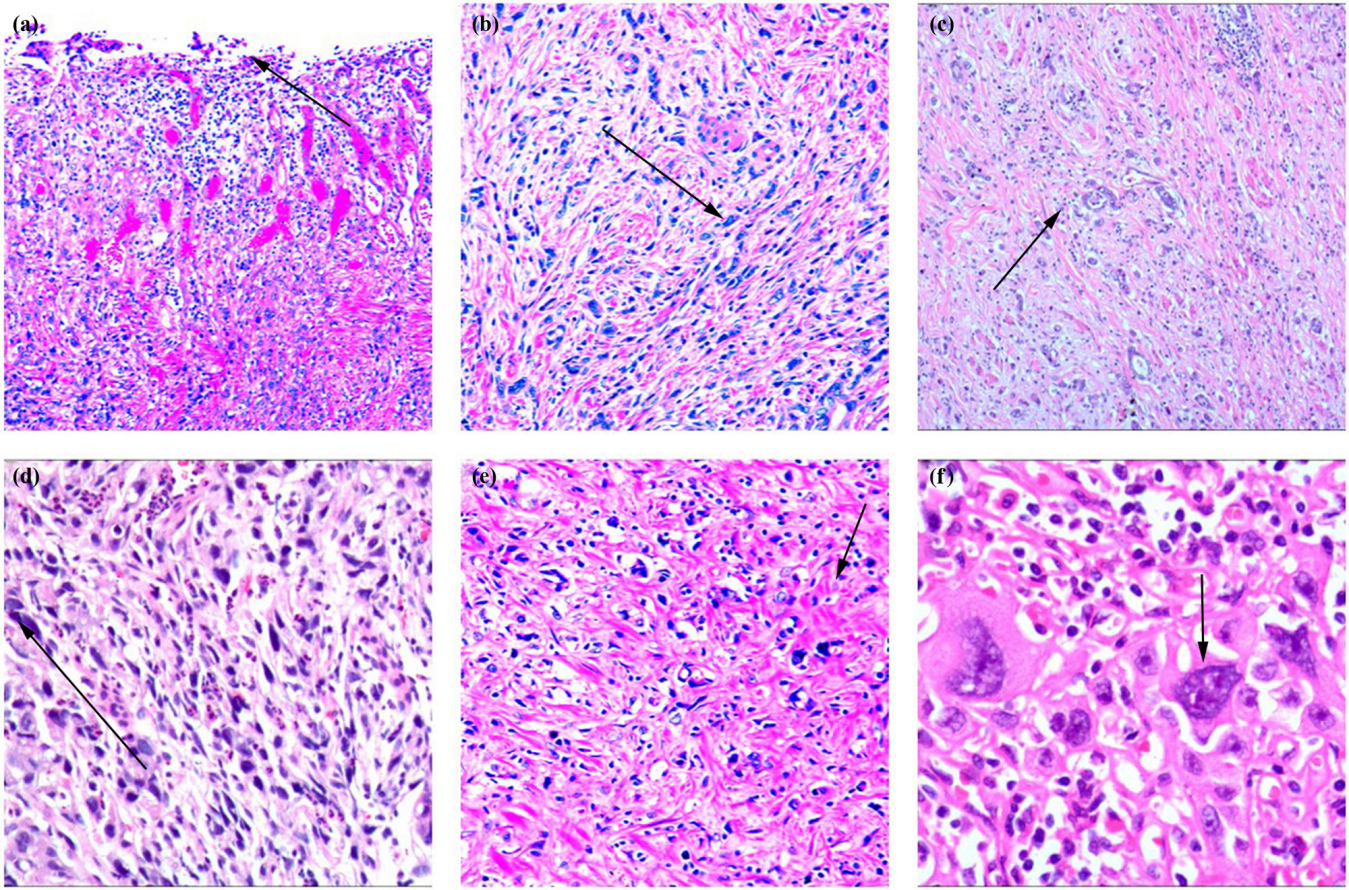
Gastric pleomorphic giant cell carcinoma: (a) histopathological features: superficial ulcers formed on the mucosa of the gastric lumen surface, and a small amount of inflammatory necrotic tissue or granulomatous tissue proliferation consisting of proliferating small blood vessels and inflammatory cells was observed (HE ×100); (b) the tumor tissue was mainly a mixture of pleomorphic spindle cells and giant cells with a wide variety of high-, intermediate-, and low-differentiated adenocarcinoma cells forming an interlocking arrangement (HE ×100); (c) spindle cells: the tumor cells were spindle-shaped and resembled a histomorphological class of sarcoma-like structures (HE ×100); (d) cytologically, they could have a short spindle shape, spindle shape, epithelioid shape, irregular shape, etc.; oddly shaped tumor cells could be observed among the spindle cells (HE ×200); (e) pleomorphic giant cell component with smooth muscle tissue of the myocardium of the gastric wall divided into nested masses of variable size or disorganized lamellar structures (HE ×200); and (f) the tumor cells were irregularly round or oval, with little cytoplasm, and basophilic, while the nuclei were irregularly round, oval, or polygonal, with oddly shaped mononuclear or multinuclear. The significant heterogeneity of multinuclear giant cells is one of the characteristics of gastric pleomorphic giant cell carcinoma (HE ×400).

Superficial ulcers were formed on the mucosa of the gastric lumen surface, and a small amount of inflammatory necrotic tissue or granulomatous tissue proliferation consisting of proliferating small blood vessels and inflammatory cells was observed (Figure 1a). The tumor tissue was mainly composed of a mixture of pleomorphic spindle cells, giant cells, and adenocarcinoma cells of various histomorphological structures of high, intermediate, and low differentiation in an interlocking arrangement (Figure 1c). The morphology of the pleomorphic spindle cells resembled that of spindle cell sarcoma (Figure 1b), which has a complex morphological presentation; the cells presented with a short spindle shape, spindle shape, irregular shape, or epithelioid shape. Cytologically, several oddly shaped tumor cells were observed among the spindle cells (Figure 1d). Pleomorphic giant cells appeared as a single giant cell among the pleomorphic spindle cells and a variety of adenocarcinomas or in a sheet-like distribution (Figure 1e).

Cytologically, the giant cells could be divided into mononuclear and multinucleated cells. Both the mononuclear and multinucleated giant cells were primarily characterized by the heterogeneity or diversity of nuclear morphology and were irregularly round or oval, with variable cytoplasmic content. Meanwhile, the nuclei were variable in size and morphology, with some being long, polygonal, irregularly round, or oval and others presenting with notably odd shapes. The nuclear chromatin of the giant cells was aggregated into a granular or cinder-block shape, and the nuclear membrane was thick and clear. It showed either large nucleoli or no nucleoli.

**Figure 2:**
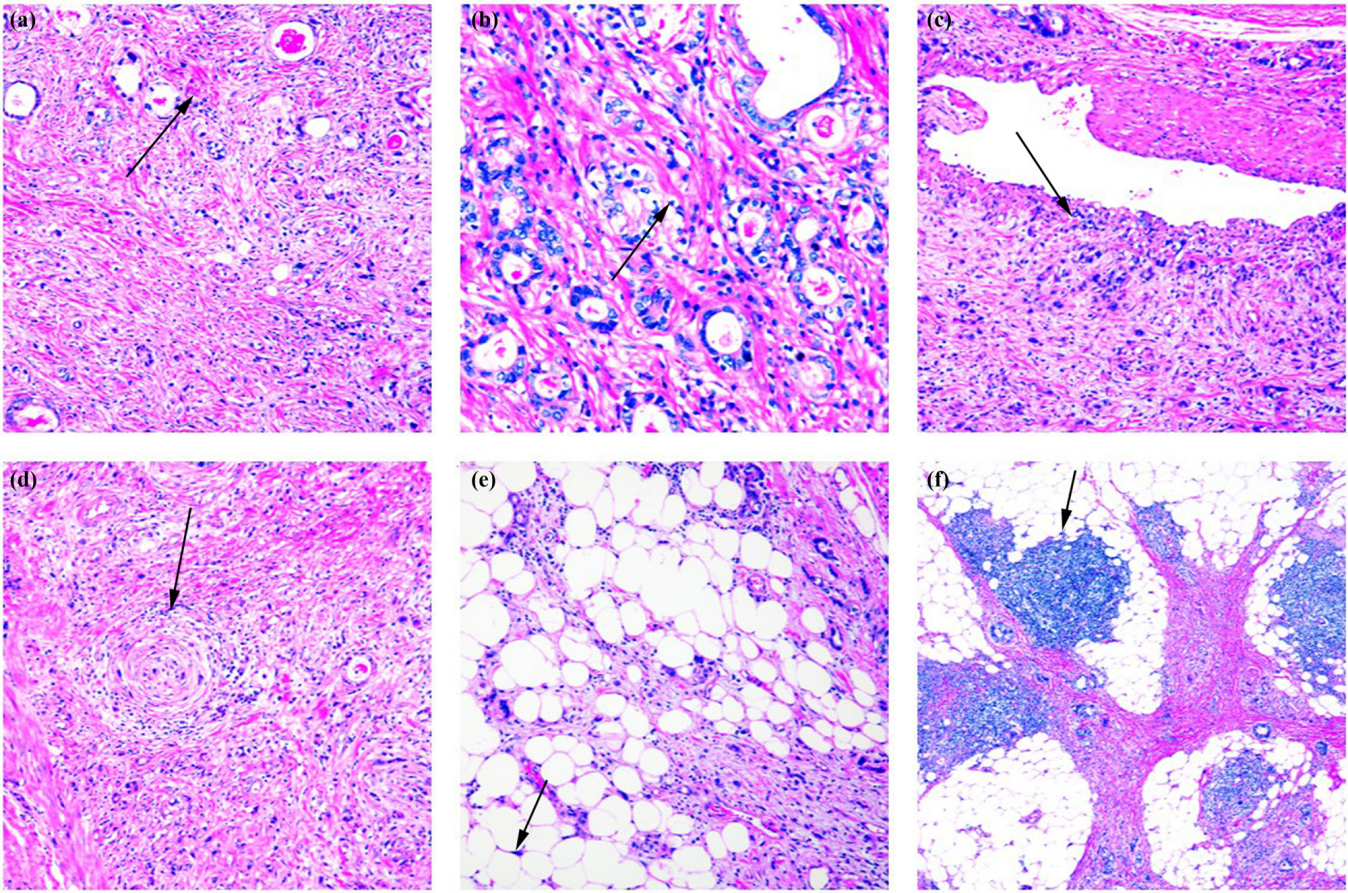
Gastric pleomorphic giant cell carcinoma: (a) a wide variety of high-, intermediate-, and low-differentiated adenocarcinomas mixed with pleomorphic spindle cell and giant cell components that were mutually interspersed (HE ×100); (b) clear cell carcinoma rich in glycogen (HE ×200); (c) invasion of tumor tissue into the blood vessels, indicating that most of the vessel walls were involved (HE ×100); (d) tumor tissue invading nerves (HE ×100); (e) the tumor tissue invading the extra serosa adipose tissue, widening the serosa, and dividing the adipose tissue into irregular pieces of fat; the tumor tissue continued to grow in a crab-foot-like pattern among the adipose tissue beyond the serosa of the gastric wall (HE ×100); and (f) the formation of massive lymphoid tissue with follicle-like structures (HE ×100).

The giant cells could be classified into small, medium, and large giant cells, with volume sizes of <40, 41–60, and >61 μm, respectively (Figure 1f). A variety of histomorphological adenocarcinoma structures were defined as tubular, papillary, lamellar, or nested masses without tubular-like structures in terms of three degrees of differentiation (Figure 2a). The histological types included common papillary, tubular, low-adherent, undifferentiated carcinomas, and rare histological types of gastric cancer, such as clear cell carcinoma rich in glycogen (Figure 2b), micropapillary carcinoma, hepatocellular adenocarcinoma, and mucinous epidermis-like carcinoma.

The tumor growth pattern, with pleomorphic giant cell carcinoma growing invasively toward the muscular layer of the gastric wall and outside the serosa with the smooth muscle fibers divided into irregular pieces, was highly penetrating and destructive. The tumor tissue formed sheet-like necrosis and destroyed the vascular wall (Figure 2c) to form vascular cancer thrombus and lymphatic vessel cancer thrombus, and the nerve invasion was often observed (Figure 2d). The tumor tissue invaded the extra serosa adipose tissue and widened the serosa; meanwhile, crab-foot-like pattern among the adipose tissue was formed for the adipose tissue dividing into irregular pieces of fat adjacent to the gastric wall (Figure 2e). There was a large amount of lymphoid tissue proliferation and lymphoid follicle formation in the peripheral area of the tumor, especially the tumor invaded the adipose tissue in the peripheral area (Figure 2f). The quantification of lymphocyte proportion is carried out below.

### 4.3 Routine immunohistochemical staining results

There was a positive expression of CKpan, CEA, CDX2, and villin; SMA and Calponin were focal expressed (Table 2).

**Table 2:** Immunohistochemical markers in seven cases

Case	Age	Gender^*^	CKpan	CEA	Villin	CDX2	p53	Vimentin	SMA	Calponin	MLH1	MSH2	PMS2	MSH6	ki-67 (%)
1	54	M	+	+	+	+	+	focal+	focal+	focal+	+	+	+	+	80
2	76	F	+	+	+	+	+	focal+	focal+	focal+	+	+	+	+	50
3	68	M	+	+	+	+	+	focal+	focal+	focal+	+	+	+	+	70
4	66	F	+	+	+	+	+	focal+	focal+	focal+	+	+	+	+	60
5	55	M	+	+	+	+	+	focal+	focal+	focal+	+	+	+	+	75
6	72	M	+	+	+	+	+	focal+	focal+	focal+	+	+	+	+	65
7	46	F	+	+	+	+	+	focal+	focal+	focal+	+	+	+	+	60

^*^M: male F: female.

Gastric pleomorphic giant cell carcinoma shows positive immunostaining for CKpan, EMA, villin, CDX2, E-cadherin, and p53. Focal expression of vimentin, Calponin, SMA, Nestin, S-100, CD99, desmin, and CD34 is observed in spindle and giant cells; Expression of Ki-67 positive cells is 70–80%.

**Figure 3:**
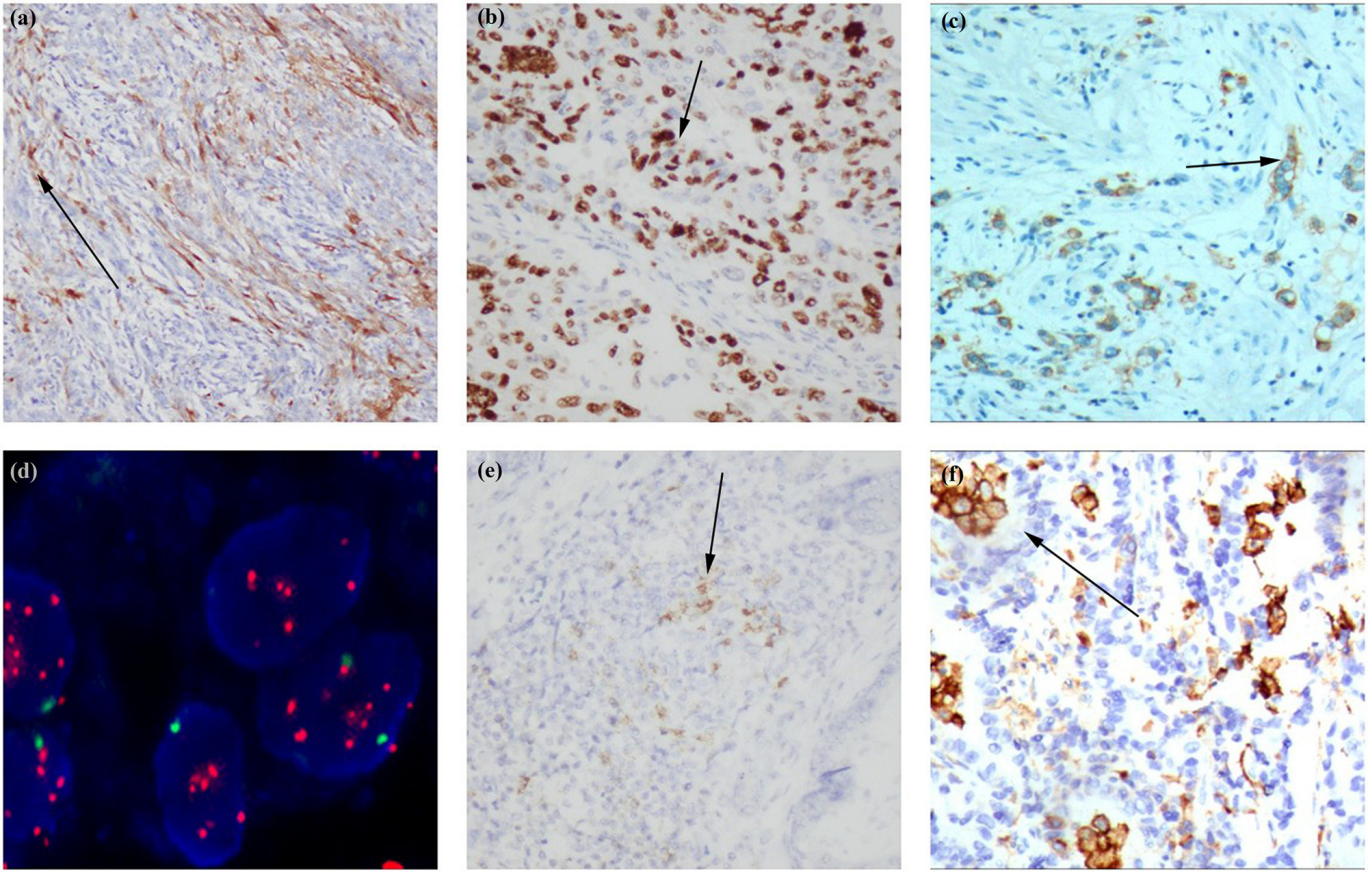
Gastric pleomorphic giant cell carcinoma: (a) focal positive expression of spindle-shaped fine calponin using the EnVision method (×200); (b) an 80% expression of Ki-67-positive cells (EnVision method, ×400); (c) the HER2 gene was amplified in large particles; the red denotes the probe signal and the green chromosome 17 (FISH method); (d) the HER2 gene was amplified in a cluster shape; the red denotes the probe signal and the green chromosome 17 (FISH method); (e) interstitial lymphocytes indicating PD1-positive expression (EnVision method ×200); and (f) PD-L1-positive expression (EnVision method, ×400).

### 4.4 Human epidermal growth factor receptor-2 gene amplification and protein expression rate

Positive HER2 protein expression was localized to the cell membrane, with three focal expression IHC3+ cases (Figure 3c), accounting for 42.9% (3/7). The HER2 gene amplification rate detected using the FISH technique was 42.9% (3/7), HER2/CEP17 ≥2.2, including one case of large granular amplification and two cases of dot amplification (Figure 3d), indicating FISH+.

### 4.5 Programmed death-1 and programmed death ligand-1 expression results

There are two cases in which the number of TPS-positive cells ≥1%, indicating PD-L1 positive. Moreover, CPS ≥1 can be considered PD-L1 positive in gastric tumor tissue. Of the seven cases in this study, three cases expressed CPS ≥1 (CPS = 8, CPS = 13, CPS = 20). In summary, PD-L1-positive cells were expressed in the gastric cancer tissues as a multifocal, patchy pattern (Figure 3e), with the positive rate being 42.9% (3/7).

Meanwhile, the positive PD1 expression in the tumor mesenchymal tissue lymphocytes was characterized by single scattered or sheet-like aggregates (mainly located in focally distributed lymphocyte aggregates among adenocarcinoma cells) (Figure 3f).

### 4.6 Follow-up results

The follow-up duration was 6–18 months, and all patients remained alive and well.

## 5 Discussion

Pleomorphic giant cell carcinoma is a rare epithelial malignancy. A growing body of literature has reported that pleomorphic carcinoma originates from a monoclonal allogeneic stem cell that forms definite cancerous tissue during growth; however, at a certain stage, cancer cells undergo transformation, leading to the appearance of spindle cells and giant cells, but the nature of these cells remains cancerous. Because under the light microscope, it has been observed that there is a reciprocal migration between cancer cells and spindle cells, and immunohistochemical studies confirmed that spindle cells and giant cell components expressed both keratin and vimentin [3,28,29]. Currently, the percentage of giant cells in pleomorphic giant cell carcinoma was inconsistent with the findings ranging from 10% [30], 40% [31], 50% [4], and more than 50% [29] to 10–100% [32].

In the present study, seven patients with pleomorphic giant cell carcinoma were assessed, and it was found that the carcinoma was mainly reflected in the pleomorphism of the histological structures. In histopathological terms, this included the pleomorphism of the cell morphology and histological structure, meaning that the proportion of giant cells was sufficient for diagnosis when it was more than 10% of the tumor cells.

The HER2 gene status will guide the targeted therapy for gastric carcinoma. Therefore, accurate HER2 assessment is crucial for patients who opt for targeted therapy. At present, the HER2 gene amplification in gastric carcinoma was a regular test in pathology departments, and HER2 detection guidelines have been formulated in many countries [33]. Gastric pleomorphic giant cell carcinoma is characterized by a focal high expression of HER2 protein and HER2 gene amplification. In recent years, immune co-stimulatory molecules have become a major focus of immunological research. Here, PD1 and its ligand, PD-L1, are deemed to play an important role in tumor progression, which are type I transmembrane proteins, consisting of 288 and 290 amino acids, respectively [34]. As noted, gastric pleomorphic giant cell carcinoma is characterized by weakly positive. MMR genes indicating genetic susceptibility were indicators of hereditary nonpolyposis in colorectal cancer. Mutations in any of the genes would result in defective cellular MMR functioning, leading to genetic instability manifesting with replication errors or microsatellite instability, thus enhancing the predisposition to tumor development. The MMR gene proteins, i.e., MLH1, MSH2, PMS2, and MSH6, are positive in gastric pleomorphic giant cell carcinoma.

The giant cells of gastric pleomorphic giant cell carcinoma have specific characteristics. The giant cells were irregularly round or oval, with little cytoplasm, and basophilic; the nuclei were long, polygonal, irregularly round, or oval, or oddly shaped with mononuclear or multinuclear. The significant heterogeneity of multinuclear giant cells is one of the characteristics of gastric pleomorphic giant cell carcinoma. The nuclear chromatin of pleomorphic giant cells is aggregated into a granular or cinder-block shape, and a thick nuclear membrane is visible. Nucleoli can be present or not present, and large odd mitotic figures are also generally observed. Meanwhile, the sizes of the giant cells vary greatly. In the present study, the giant cells were divided into small, medium, and large cells based on the volume sizes of <40, 41–60, and >61 μm (measured by mirror table micrometer [table ruler] under the microscope), respectively. The characteristics of fusiform cells in gastric polymorphic giant cell carcinoma, the histological morphology of fusiform cells is not single, and the diversity of fusiform cell structure is arranged in the block structure. The complex shape of spindle-shaped cells is one of the characteristics of gastric polymorphic giant cell carcinoma [18].

The spindle cells of gastric pleomorphic giant cell carcinoma are characterized by the non-uniformity of the histological morphology of spindle cells, with a diverse spindle cell structural arrangement forming a lamellar structure. This complex morphological presentation is characteristic of this type of carcinoma, i.e., a histomorphological class resembling sarcoma or with sarcoma-like mesenchymal structures, generally observed as a mixture of spindle cell sarcoma, spindle cell carcinoma, spindle cell mesenchymal sarcoma, and other structures.

Here, the following histopathological diagnostic points for pleomorphic giant cell-type gastric carcinoma were proposed: (1) giant cells accounting for more than 10% of the whole tumor, with both large, medium, and small giant cells and nuclei aggregated in masses, cinder blocks, and odd shapes; (2) non-uniformity of the spindle cell morphology, with a diverse spindle cell structural arrangement forming a lamellar pattern; (3) adenocarcinoma cells with a wide variety of histomorphological structures of any proportion with high, intermediate, and low differentiation; (4) pleomorphic spindle cells and giant cells mixed with any proportion of various histomorphological structures of adenocarcinoma with high, intermediate, and low differentiation, forming an interlocking arrangement; (5) vascular tumor thrombus, lymphatic tumor thrombus, and nerve invasion easily observed, with 1–3/10 high power field in each type; (6) the proportion of cells with a positive cell proliferation index, Ki-67, accounting for 70–80%; and (7) positive HER2 protein expression and HER2 gene amplification both being focal, with a high expression of PD1 and the presence of the MMR gene proteins MLH1, MSH2, PMS2, and MSH6.

For differential diagnosis, the following standards apply: the first diagnosis is sarcomatoid carcinoma of the stomach, which is essentially derived from epithelial cells that have sarcoma cells; they are not true sarcomas but carcinomas in the form of spindle cell variants. These sarcomatoid spindle cells are still cancer cells in nature but are differentiated in terms of the direction of the sarcomatoid cells. Sarcomatoid carcinoma is also a rare tumor with a poor prognosis, primarily comprising spindle cells. It does not involve a mixture of adenocarcinoma cells with a wide variety of histomorphological structures with different degrees of differentiation of pleomorphic giant cell carcinoma. Giant cells are rarely seen [35].

The second type of cancer is mixed gastric tumors. It referred to the existing two or more histological structures in a single tumor, which can be of epithelial, mesenchymal, or lymphoid origin, with each component having a separate histological structure. The two different histological structures are generally separated by incomplete fibrous connective tissue, and their prognosis or the risk of lymph node metastasis depends on the type and number of hypo-differentiated tissue components [36]. Gastric pleomorphic carcinoma is also a high-grade mixed carcinoma of the stomach, which is called high-grade mixed carcinoma of the stomach. Mixed gastric tumors feature a diversity of histological structures, while gastric pleomorphic giant cell carcinoma is characterized by giant cell morphology.

Third, fibromatosis-like metaplastic carcinoma, which occurs mostly in the breast and rarely in the stomach, should be considered. Histologically, this form of carcinoma is composed of spindle cells accompanied by glandular, squamous epithelial, or heterologous components; the spindle cells are morphologically diverse and may exhibit mild or regional pleomorphism and possibly be arranged in fascicles. This type is commonly in areas composed of vascular luminal-like structures and areas of squamous cell differentiation. It is a low-grade saprophytic carcinoma with a histological presentation similar to that of ligamentous-type fibromatosis. It is not characterized by the small, medium, and large giant cell types of pleomorphic giant cell carcinoma or the features of these giant cells. In terms of immunophenotype, this involves tumor cells expressing CKpan and 34ßE12, while the spindle cells are positive for vimentin expression [37,38].

Primary choriocarcinoma of the stomach is rare. Gastric choriocarcinoma, a highly malignant tumor secondary to chylothorax, miscarriage, or full-term delivery, often occurs following ectopic pregnancy, mostly in women of childbearing age. It occasionally occurs in the ovaries of unmarried women and is known as primary choriocarcinoma. The attendant pathomorphology exhibits a lamellar distribution of cytotrophoblast and syncytial trophoblast cells; tumor cell heterogeneity is obvious, and large areas of necrosis and hemorrhage are generally observed. In terms of immunophenotype, the tumor cells are positive for cytokeratin (CK) and chorionic gonadotropin (hCG) expression and negative for CEA and alpha-fetoprotein, with the Ki-67-positive index is >80% [39].

Another type of differential diagnosis is gastric myeloid sarcoma, a concept introduced in *WHO Classification of Lymphohematopoietic Tumors* (2001 edition),which presents a neoplastic mass formed via infiltration of primitive or naive myeloid cells in organs and tissues other than the bone marrow [40]. There are three types of myeloid sarcoma, namely granulocytic sarcoma, monocytic sarcoma, and tumors composed of erythrocytes, leukocytes, and megakaryocytes or just erythrocytes and megakaryocytes. Myeloid sarcomas that occur in the stomach are rare, especially those composed of megakaryocytes. The histology presents predominantly diffuse or striated, columnar arrangement with infiltrative growth. In terms of immunophenotype, it showed positive expression of CD33, CD34, CD43, lysozyme, MPO, CD163, CD117, CD3, and CD20 [41].

The sixth diagnosis is gastric compound large-cell neuroendocrine carcinoma, which is rare and has been reported as occurring mainly in the lungs. Histologically, the tumor cells are large, with a moderate to abundant cytosolic mass and distinct nuclei. They are arranged in organoid nests and rosette forming, often with several tubular adenocarcinoma tissues and squamous cell carcinoma tissues. Adenocarcinoma tissue does not have the diverse histomorphological structures of high, intermediate, and low differentiation of pleomorphic giant cell carcinoma, nor does it have the small, medium, and large giant cell types of pleomorphic giant cell carcinoma or the characteristics of these giant cells. In terms of immunophenotype, the tumor tissues express chromogranin A (CgA), synaptophysin (Syn), and CD56. Squamous cell carcinoma tissues express CK5/6 and p63, and adenocarcinoma tissues express CK7; villin and CDX2 are not negative in this tissue type [42].

The last differential diagnosis is gastric ulcer/carcinoma of gastric ulcer/granulomatous inflammation formed by a gastric ulcer showing many giant cells. Here, the lesions can present with a diverse spindle cell morphology and may exhibit mild/or regional multinucleated giant cells, with areas of vascular luminal-like structures commonly observed. It manifested as inflammation-like gastric ulcer rather than diverse histomorphological structures with the high, intermediate, and low differentiation of gastric pleomorphic giant cells. They also do not have the small, medium, and large giant cell types of pleomorphic giant cell carcinoma or the characteristics of these giant cells [43].

In conclusion, In this study, we summarized the histopathological diagnostic points for pleomorphic giant cell-type gastric carcinoma; besides, we analyzed the differential diagnosis for gastric pleomorphic giant cell carcinoma while clarifying the histopathologic features of this type of tumor. However, our study has limitations too.

## 6 Conclusion

Our study found that gastric pleomorphic giant cell carcinoma is rare. In this study, based on the morphological and histological characteristics of cancer cells, gastric pleomorphic giant cell carcinoma is defined as: pleomorphic spindle cells and giant cell components occupy the entire more than 10% of the tumor, while the pleomorphic spindle cell and giant cell components and any proportion of highly, moderately, and poorly differentiated adenocarcinoma components of a variety of histological and morphological structures are mixed and arranged in a staggered manner. Gastric pleomorphic giant cell carcinoma in addition to the pleomorphism of spindle cells, especially, emphasizes the pleomorphism of giant cells and has small, medium, and large giant cell types and morphological characteristics. Gastric pleomorphic giant cell carcinoma has a variety of histomorphological features and is easily confused with a variety of tumors; clarifying the histopathological features of the tumor is of great significance for differential diagnosis and precise treatment.

In this study, we summarized the histopathological diagnostic points for pleomorphic giant cell-type gastric carcinoma based on the morphological and histological structural characteristics of cancer cells. Besides, we analyzed the differential diagnosis for gastric pleomorphic giant cell carcinoma. Gastric polymorphic giant cell carcinoma, in addition to the polymorphic spindle cells, emphasizes the polymorphic giant cells, and has small, medium and large giant cell types and morphological characteristics. Gastric pleomorphic giant cell carcinoma demonstrates a variety of histological features, which can be easily confused with various tumors. Clarifying the histopathological characteristics of this tumor is of great importance for differential diagnosis and precise treatment. Studies in biology, genetics and etiology, as well as differences between abnormal protein expression and clinicopathological parameters, need more cases to accumulate, longer follow-up and better molecular biology studies.
